# Patient satisfaction with remote monitoring of cardiac implantable electronic devices: the Valiosa questionnaire

**DOI:** 10.1186/s12913-020-05216-3

**Published:** 2020-04-25

**Authors:** Miguel A. Ruiz Díaz, Marta Egea García, Roberto Muñoz Aguilera, Xavier Viñolas Prat, Jorge Silvestre García, María Álvarez Orozco, José Martínez Ferrer, On behalf of the VALIOSA study group, Socorro Sorbet, Julia Díaz, Celia Hijosa, Carmen Varela, Francisco Méndez, Olga Calvo, Omar Al-Rizzo

**Affiliations:** 1 Department of Methodology, School of Psychology, Universidad Autónoma de Madrid, Madrid, Spain; 2 Health Economics & Outcomes Research Department, Medtronic Ibérica, S.A, Madrid, Spain; 3 Department of Cardiology, Hospital Universitario Infanta Leonor, Madrid, Spain; 4 Department of Cardiology, Hospital Santa Creu i Sant Pau, Barcelona, Spain; 5 Department of Cardiology, Hospital Universitario La Paz, Madrid, Spain; 6 Department of Cardiology, Hospital Universitario de Araba, Vitoria, Spain

**Keywords:** Patient satisfaction, Patient reported outcomes, Remote monitoring, Cardiac implantable electronic devices, Patient health questionnaire

## Abstract

**Background:**

Remote monitoring of cardiac implantable electronic devices (CIEDs) has demonstrated substantial benefits. Treatment guidelines have therefore endorsed its use and is being increasingly adopted in the clinical setting, but the level of satisfaction they convey remains still unknown. We developed and validated a questionnaire to measure patient satisfaction with remote monitoring using Medtronic CareLink® Network and assessed its internal reliability and dimensional validity.

**Methods:**

After a thorough literature review, cognitive debriefing of 18 patients, and an expert panel discussion, a 30-item instrument was proposed and grouped into 5 dimensions (items): 1- Information on cardiac condition (3), 2- Device convenience (3), 3- Transmission process (6), 4- Satisfaction with medical monitoring (8), and 5- General opinions (10). Correlation with the visual analog scale (VAS), overall health related quality of life (HRQoL) measured by the EuroQoL-5D accompanied by the VAS as well as with the Medical Outcomes Study (MOS) SF-36 were assessed. Psychometric properties, exploratory factor analysis and a second order confirmatory factor analysis (a hierarchical CFA with a general common factor explaining the relations between the first order common factors, See Figure 1) were estimated. Models were assessed based on item loading size, sign and statistical significance, and goodness-of-fit statistics.

**Results:**

A total of 186 patients (77% male) with a mean age of 66.03 (SD = 13.94) years were assessed. 48% had implantable cardioverter-defibrillators, 24% had pacemakers, and 29% had cardiac resynchronization therapy devices. An overall Cronbach’s α = 0.893 was achieved, with acceptable reliabilities for isolated dimensions. Correlations with corresponding VAS scales were meaningful and significant (*p* < 0.01). The second order factor solution yielded good goodness-of-fit indexes (χ^2^/df = 1.44, CFI = 0.96, TLI = 0.95, RMSEA = 0.05). Satisfaction with remote monitoring was not related to HRQoL *(r* < 0.05), except for the correlation between the SF-36 mental component and the information on cardiac condition dimension (*r* = 0.263, *p* < 0.001).

**Conclusions:**

The 30-item questionnaire showed good reliability and validity to assess satisfaction with remote monitoring in patients with CIEDs.

## Background

Technology available for managing cardiac implantable electronic devices (CIED) has advanced considerably. In this sense, remote monitoring (RM) represents a complement to routine in-office care that provides access to a tremendous wealth of information recorded and stored by CIEDs such as device performance, including history of cardiac arrhythmias, battery and lead parameters without face-to-face interaction. Remote monitoring enables remote feedback from device to physician and allows for continuous remote monitoring, which improves patient safety and care by allowing any device problems to be detected immediately [1]. In addition to clinical benefits, RM has been shown to allow for longer intervals between in-office visits and a shorter visit duration as querying the device is no longer necessary, thus lightening the burden of in-hospital follow-up of patients with implanted devices, with the consequent cost reduction [2, 3] Thanks to demonstrated substantial benefits, treatment guidelines have endorsed its use for all eligible patients and RM is being increasingly adopted in the clinical setting [4].

Despite the existing clinical and economic evidence available, patient reported outcomes have received little attention, even though they are expected to play a more prominent role in assessing performance and determining the comparative effectiveness of treatment alternatives, in part because of a growing emphasis on patient-centered care and value-based procurement initiatives.

In particular, patient satisfaction represents an important measure of the extent to which a patient is satisfied with all aspects of healthcare delivery that are of relevance to health, which could be related with the quality of care patients receive. It has been shown that patient satisfaction affects patients’ health-related decisions and treatment-related behaviors (like appropriate use of services, correct medication use or treatment continuation), and consequently, impacting substantially the success of treatment outcomes [5].

To date, few publications evaluating patient satisfaction associated with RM of patient with CIEDs have been published. Large registries like ALTITUDE and PREDICT-RM did not include patient reported outcomes [6, 7]; besides these registries, certain observational studies have considered measures of health status, satisfaction and/or acceptation, remote patient monitoring experiences, and preferences for follow-up but using a self-designed questionnaire that did not undergo a strict validation process [8–11]. There is currently a European randomized controlled trial ongoing (REMOTE-CIED) designed to examine the patient perspective of RM but it only covers patients with implantable cardioverter-defibrillators (ICD) and cardiac resynchronization therapy devices (CRT) [12].

Despite the increasing interest in patient opinions and concerns, to date there is no validated questionnaire specifically designed to measure patient satisfaction with CIED RM systems. We believe that patient satisfaction measurement is not only a compromise with quality management for health service providers [13] but also a need towards identifying problematic users, and particularly among those who do not make automated transmissions (like pacemaker users).

The aim of the present study was to develop a new questionnaire with proven psychometric properties that measures patient satisfaction with RM in patients with CIEDs, focusing on a specific RM system, CareLink® Network (Medtronic, Minneapolis, MN), currently used by more than 1,300,000 patients from more than 1200 health centers worldwide in about 80 different countries [14].

## Methods

The present research was designed as an observational, two-stage, cross-sectional, multicenter study to develop a specific instrument for the measurement of patient satisfaction with RM of CIED. Patients with implanted pacemakers (PM), ICD, and CRT devices who had been using CareLink® Network for at least 2 months before recruitment were included. The study was conducted under actual treatment conditions for their disorder in clinical practice.

The Medtronic CareLink® Network is the Internet-based remote monitoring service for patients with Medtronic implanted cardiac devices. It allows patients a convenient timely connection to their clinic using the person’s monitor/ smartphone/ tablet to collect and transmit device data. A hand-held smart reader is used to collect data from the implanted device. The smart reader communicates with the monitor and, through a software application, it transfers the data to the CareLink® Network. All the information is recorded and reviewed by the nurses through the online platform. In case there is a critical event, the patient is called by the medical staff to visit the physician (Figure 6 Additional file 3).

The study was performed following the Helsinki-Tokyo-Venice guidelines for human research.

### Development phase: study design and participants

The aim of the development phase was to create and design the VALIOSA questionnaire incorporating the patient perspective.

For that purpose, a thorough literature review and patient focus groups were conducted to identify relevant contents and topics addressing treatment satisfaction and patient management.

Two focus groups were held at two different hospitals. The first one took place at Hospital Universitario de Araba (Vitoria) with 10 patients and the other one at Hospital Infanta Leonor (Madrid) with 8 patients. Patients were recruited at random from those with a scheduled visit in the selected week and balancing for gender and age. The topics discussed included symptoms and disease burden, daily limitations, device-related discomfort, lifestyle changes, impact on social relationships, family and specialized support, and appropriate follow-up. Patient contributions were summarized following a semantic reduction process by two independent content specialists. As a result, a preliminary questionnaire of 52 items was proposed.

This raw version underwent a discussion and semantic refinement process by an expert panel consisting of four cardiologists, one psychometrician and one pharmacist. Redundant and inappropriate items were discarded and replaced by equivalent items and was reduced to 37 items.

This initial set of 37 items comprised the following RM aspects: information on monitor use, training and convenience of monitor use, problems with the transmission process, satisfaction with medical care and RM, and benefits and impact on activities of daily living.

The reviewed version was administered to a pilot sample consisting of 10 patients to check comprehension and acceptance, and the information gathered was used to refine the items. The resulting version was composed of 30 items grouped into 5 dimensions (number of items): information (3), monitor convenience (3), monitor handling and transmission process (6), disease follow-up (7), general impression and benefits (11). A panel composed by 9 specialists (cardiologists, nurses and psychometricians) assessed content validity, scoring item adequacy for measuring each content dimension and the unidimensional [15] and multidimensional [16] item-domain congruence indexes were computed.

### Validation phase: study design and participants

The aim of the validation phase was to administer the 30-item version to a representative sample of patients to check item fit to the proposed construct, check psychometric properties of the preliminary questionnaire designed in the development phase, and reduce the number of items (if necessary) in accordance with the proposed construct.

### Patients

Three samples of patients were recruited at 4 hospitals in 3 regions of Spain. A pilot sample of 10 patients was recruited to check item comprehension and to identify possible response problems. Finally, a sample of 186 patients was recruited to measure psychometric properties. All patients had to meet selection criteria: being older than 18 years of age, being implanted with a CIED, having more than 2 months of experience using RM, being able to understand and answer questionnaires in Spanish, and having no cognitive impairment.

Sample size was determined based on the anticipated number of dimensions, the number of items per dimension (3:1) [17, 18], the number of patients per item (4:1), [19, 20] and the sample representativeness (*n* > 150) [21]. A sample size of 180 patients with complete information was deemed necessary.

### Psychometric properties of final version

Dimension and overall scale reliability (internal consistency) was estimated by computing Cronbach’s alpha and 95% confidence interval intra-class correlation coefficient (ICC) for internal consistency.

To test the construct validity of the questionnaire, a confirmatory factor analysis was performed including all items, assuming a second order factor structure with items as indicators of the proposed first order dimensions and with all dimension loading in an overall satisfaction second order dimension. The robust weighted least squares estimation method was used.

The following psychometric properties were studied. Feasibility: response time, floor-ceiling effect, and missing values distribution; Internal Consistency/Reliability: Cronbach’s alpha, item correlations, adjusted item-total correlation, ICC and omega [22]; Construct Validity: Confirmatory factor analysis; Concurrent Validity: Correlation between the new questionnaire (Valiosa) and the VAS measures; Convergent and construct Validity: Correlation between similar/dissimilar dimensions of the Valiosa, MOS SF-36 dimensions and corresponding VAS measures.

### Instruments

The final refined questionnaire, named Valiosa (see Additional files 1 & 2), was included in a clinical record form along with questions about patient social and demographic characteristics, relevant clinical information, the MOS SF-36, and 6 VAS.

The Valiosa questionnaire is a 30-item, self-completed questionnaire measured on a 5-point Likert scale (0 = No, nothing, 4 = Yes, a lot) to measure patient satisfaction with RM. It is structured in 5 dimensions: 1- Information on cardiac condition (3 items), 2- Device convenience (3 items), 3- Transmission process (6 items), 4- Satisfaction with medical monitoring (8 items), and 5- General opinions (10 items). Dimension scores are obtained by adding item scores for that dimension and transforming the obtained score to a 0–100 common metric. The rescaled score is obtained using the following transformation:

$$ Y\hbox{'}=\frac{Y_{obs}-{Y}_{\mathrm{min}}}{Y_{\mathrm{max}}-{Y}_{\mathrm{min}}}\times 100 $$

Where Y_max_ = maximum dimension score, Y_min_ = 0 (minimum score dimension), Y_obs_ = observed dimension score (algebraic summation), and Y′ = transformed score. Scores for items 9, 10, 12, and 19 need to be reversed before summation. An overall score can be calculated by averaging the dimension scores.

Additionally, five 15-cm VAS (0 = Completely unsatisfied, 10 = Completely satisfied), with 10 marks along the scale, were used as convergent measures of the same concepts contained in the dimensions. The HRQoL VAS accompanying the EuroQoL-5D [23] was used to measure overall health related quality of life and as a distinct measure from satisfaction. HRQoL was also measured using the MOS SF-36 [24]. This is a generic instrument measuring 8 dimensions arranged in 2 summary components (physical and mental) using 36 items.

All analyses were performed using IBM SPSS 20, AMOS 20, and Mplus 7.31 software.

## Results

### Development phase

#### Focus groups

Of the 18 participants in the 2 focus groups, 78% were male, 56% had PM, and 44% had ICD. Mean age was 67.5 years (SD = 7.1, min. = 54, max. = 80), mean time since implantation was 2.03 years (SD = 5.8, min. = 2 months, max. = 20 years), and mean use of CareLink® Network was 2.03 years (SD = 1.7, min. = 2 months, max. = 5 years).

Most patients reported living a normal life for their age, although they tried to avoid physical exertion. Some reported they had adopted healthier habits (no smoking or alcohol consumption) since diagnosis, although they thought that other diseases (e.g., diabetes) need more stringent care.

PM patients showed some distrust of the transmission process. They wished they could have made the first transmission under supervision at the hospital. One patient called in 20 min after each manual transmission, even if the modem beeped at the end. ICD patients felt they had tight supervision by their nurse, even receiving phone calls when transmission errors occurred or when fluid retention was observed during regular monitoring.

Intervals between face-to-face visits were perceived as too long. One patient visited the medical team bringing sweets to keep in touch. Self-support groups were not welcome, only the clinician was trusted for delivering information. Patients did not manifest any other needs or concerns and the saturation for information was reached in each focus group.

### Validation phase

#### Sample description

A sample of 186 patients was recruited between 2014 and 2015; 53% at Hospital Universitario de Araba, 30% at Hospital Infanta Leonor, 15% at Hospital Sant Pau, and 3% at Hospital Universitario La Paz. On average, patients were 66.03 (SD = 13.94) years old, 77% were men, 48% had ICD, 29% had PM, and 24% had CRT devices. Nearly 77% were experienced in the use of CareLink® Network, and 23.3% were naïve to this RM system. Other descriptive variables are presented in Table 1.

**Table 1:** Sample description of social/demographic and clinical variables

Employment Status (%)		Marital Status (%)	
Working	13.6	Single	9.5
Homemaker	8.9	Married	75.0
Retired	59.2	Divorced	6.0s
Unemployed	2.4	Widowed	8.3
Disabled	16.0	Other	1.2
Education Level (%)		Clinical (%)	
None	8.1	ICD	47.8
Primary	45.3	PM	28.6
Secondary	12.2	CRT	23.6
Professional Degree	16.9		
Higher	17.4		
Risk Factors (%)		Heart Disease	
Hypertension	59.5	Ischemic	53.4
Hypercholesterolemia	53.7	Dilated cardiomyopathy (non-ischemic)	33.6
History of AFib	38.8	Valvular	6.9
Smoking	37.2	Brugada syndrome	2.3
Diabetes	28.1	Other	6.1
History of renal insufficiency	17.4	None	3.8
History of sudden death	8.3		
Stroke	8.3		
CareLink use (%)		Sex (%)	
Experienced	76.7	Women	23.0
First implantation: Naïve	21.0	Men	77.0
Replacement/upgrade: Naïve	2.3		
Sample size (N)	186		

*ICD* Implantable Cardioverter-Defibrillator, *PM* Pacemaker, *CRT* Cardiac Resynchronization Therapy device

### Acceptance and responsiveness

Most patients (78.5%) fully understood all items and left no blank responses, while 14.5% left one item blank and 3.8% left two. Two patients were excluded, because they did not answer any question or left more than 80% blank. Items with the highest non-response rates were item 11 (13%) about getting support for technical problems and item 12 (5%) about having trouble on holidays. We assumed that these items were left blank due to inapplicability. The full response rate was slightly lower (75.8%) for the MOS SF-36.

Almost all items presented a unimodal skewed distribution of responses across response categories with a marked ceiling/floor effect (more than 50% of responses in the extreme category), and only two items did not obtain responses in both range extreme categories. Dimension average scores ranged within 82.0 and 91.4 with a total average of 88.8 (see Table 2). Differences between mean dimension scores were statistically significantly (*p* < 0.05), except for Disease Information vs. Convenience (*p* = 0.97) and vs. Monitoring (*p* = 1.0).

**Table 2:** Summary and reliability estimates of the Valiosa questionnaire by dimension

Dimension	Initial number of items	Mean	Standard Deviation	Cronbach’s Alpha	95% ICC		Omega	Items
					Lower	Upper		
1. Disease Information	3	91.4	13.6	.588	.473	.682	.79	1, 2, 3
2. Convenience	3	93.1	12.2	.772	.707	.824	.92	4, 5, 6
3. Transmission	6	82.0	16.1	.576	.467	.669	.76	7, 8, 9, 10, 11, 12
4. Monitoring	7	90.1	10.7	.614	.521	.695	.86	13, 14, 15, 16, 17, 18, 19
5. General Benefits	11	86.8	14.2	.836	.797	.870	.94	20, 21, 22, 23, 24, 25, 26, 27, 28, 29, 30
Total Scale	30	88.8	9.9	.893	.866	.917	.96	1–30

*ICC* Intra-class correlation coefficient

### Reliability

The overall scale reliability was good (α = 0.893, ω = 0.95) while dimension reliabilities were good for General Opinions (α = 0.836, ω = 0.94) and acceptable for Convenience (α = 0.772, ω = 0.92), Monitoring (α = 0.614, ω = 0.86), Information (α = 0.588, ω = 0.79), and Transmission (α = 0.576, ω = .76) (see Table 2). Since the scale is thought to be multidimensional, α may be underestimating the true reliability and ω values should be considered more informative.

### Validity

#### Construct validity

Content validity results showed that experts were able to identify the different domains measured but with a relative overlap between medical care and overall benefits dimensions (See Additional file 3).

**Fig. 1:**
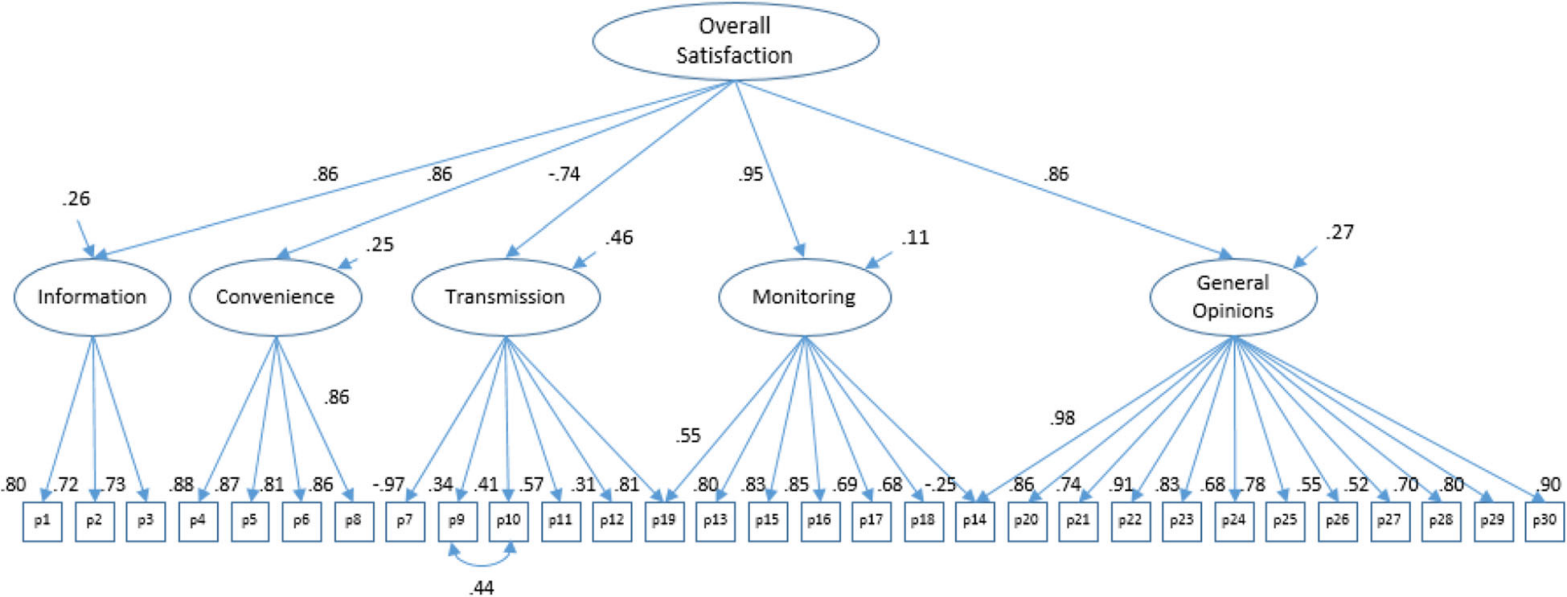
Confirmatory Factor Analysis standardized solution

Correlations between item scores fit the proposed second order factor model with an acceptable goodness-of-fit: χ^2^/df = 1.63, CFI = 0.94, TLI = 0.94, RMSEA = 0.064. All first-order loadings were significant (except for item 19) as were second order loadings (see Fig. 1). However, several modifications had to be incorporated into the model. Item 8 loaded on the Convenience dimension, item 14 showed cross-loading on the General Benefits dimension, item 19 showed cross-loading on the Transmission dimension, and items 9 and 10 exhibited a significant correlation between their error terms. After accepting these additional specifications, the model provided a substantially better fit: χ^2^/df = 1.44, CFI = 0.96, TLI = 0.95, RMSEA = 0.05.

#### Convergent validity

All dimensions significantly correlated (*p* < 0.01) with the corresponding VAS measure, although Monitoring tended to correlate more highly with Convenience, Transmission, and General Opinions, while information exhibited a similar correlation with all other VAS scales (differences in correlation below 0.110 were not statistically significant).

Correlations with divergent measures, i.e., the two MOS SF-36 HRQoL and with the HRQoL VAS measure, were negligible (Tables 3 & 4), except for the correlation between the General Opinions dimension and the HRQoL VAS measure (*r* = 0.157, *p* < 0.05); while the HRQoL VAS measure correlated more with the Physical component (*r* = 0.550) than with Mental component (*r* = 0.272).

**Table 3:** Correlations among Valiosa dimensions (rows) and visual analog scales (columns)

Dimension	VAS Measure				
	Information	Convenience	Transmission	Monitoring	General Opinion
Disease Information	.333**	.258**	.376**	.376**	.337**
Convenience	.112	.478**	.408*	.306**	.470**
Transmission	.104	.405**	.406**	.323**	.351*
Monitoring	.206**	.501**	.504**	.422**	.534**
General Benefits	.259**	.463**	.404**	.486**	.488**

*HRQoL* Health-related Quality of Life. Significance: ^*^p < 0.05, ** *p* < 0.01

**Table 4:** Correlation between Valiosa dimensions and health-related quality of life measures

Dimension	SF-36 Component		
	Physical	Mental	HRQoL
Disease Info.	.030	.263**	.094
Convenience	.110	.043	.088
Transmission	−.006	−.036	.019
Monitoring	.090	.043	.061
General Benefits	.106	.035	.157*
Physical	1.000	.012	.550**
Mental	.012	1.000	.272**
HRQoL	.550**	.273**	1.000
Mean	37.2	52.1	73.1
Standard Deviation	8.68	10.54	19.88

** *p* < 0.01, * *p* < 0.05

#### Known groups validity

**Fig. 2:**
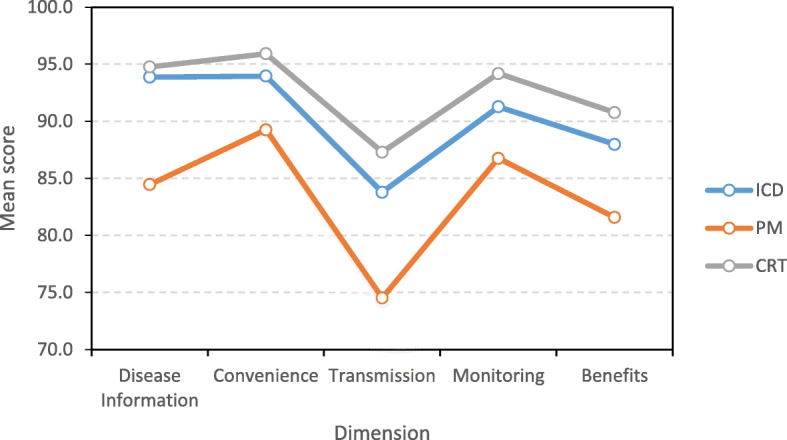
Valiosa mean dimension score by device type. Note: ICD=Implantable Cardioverter-Defibrillator, PM=Pacemaker, CRT=Cardiac Resynchronization Therapy device.

Valiosa dimensions were shown to be sensitive between different types of patients. Significant differences were found in the dimension mean scores between patients depending on the type of implanted device (F_2,179_ = 14.0, *p* < 0.001), with no interaction with the dimension score factor (F_8,716_ = 1.09, *p* = 0.368). Overall, patients with PM were significantly more dissatisfied than those with ICD (d = − 6.8, *p* < 0.001) and CRT devices (d = − 9.3, *p* < 0.001), but the latter did not differ from one another (d = 2.4; *p* = 0.477). This pattern was repeated for all dimensions (Fig. 2), except for Convenience, where ICD and PM patients did not reach significance (d = − 4.7, *p* = 0.078).

No significant differences were found in mean dimension scores between patients based on their experience with CareLink® Network (F_2,163_ = 0.77, *p* = 0.462), although initial implantation patients naïve to CareLink® Network (M = 87.2) were slightly less satisfied than experienced users (M = 89.0) and replacement patients naïve to CareLink® Network (M = 92.3).

Significant differences were found in mean dimension scores between patients from different hospitals (F_3,171_ = 3.65, *p* = 0.014) with an interaction term close to significance (F_12,684_ = 1.68, *p* = 0.068), with average satisfaction at one of the treatment sites significantly worse than the others. After excluding that site from the analysis, more statistically significant differences were found (which could be explained by additional information received from the sites).

Although small, a significant correlation was found between patient age and the Health Monitoring dimension (*r* = 0.183; *p* = 0.013), but not for the remaining dimensions nor for the overall score.

## Discussion

To our knowledge, this is the first validated questionnaire developed to measure patient satisfaction with remote monitoring of patients with CIED. This 30-item questionnaire showed good reliability and validity to assess satisfaction in patients implanted with all types of CIED, suggesting that the questionnaire is valid across different groups of patients. Patients who were naïve in the use of CareLink® Network along with those who had more than 1 year of experience were included, and this classification factor was not shown to have an influence on the level of satisfaction, suggesting that the system is well accepted from very early stages in its use. The level of satisfaction of the study population was found to be very high, with scores of more than 80 in all dimensions. Initial results were reported at the 23rd International Society for Quality Of Life Research Annual Conference [25].

### Study findings

Three items proved somewhat problematic. Item 8 (“It is easy for me to make the transmission”), was initially proposed as an indicator of the Transmission Process dimension, but it was ultimately better allocated to the Convenience dimension, and cross-loading in both dimensions was proposed. Item 19 (“The fact that the consultation is done at home instead of at the hospital is a problem for me”), initially assigned to the Monitoring dimension, showed significant cross-loading with the Transmission dimension. Item 14 (“Using the transmitter makes me feel better cared for”), initially assigned to the Monitoring dimension, showed significant cross-loading with the General Benefits dimension. Nevertheless, moving these items to the dimensions they cross-load with does not improve dimension reliability, suggesting they should remain in their proposed dimensions pending on other additional evidence. While the overall scale exhibited good reliability, some individual subscales showed only acceptable reliability when accounting for the multidimensional structure, suggesting that they should be used isolated with care.

The second order factor structure did fit our data properly. Items (first order indicators) loaded with a statistically significant weight and most with a loading (in absolute value) above 0.6. The exception was the relatively low loading for items 9 and 10, which also showed correlation between their error terms. This behavior could be associated with a response bias for PM users, since they transmit only once a year, and most did not have the opportunity to experience any transmission problem. First order dimension loadings on the Overall Satisfaction second order dimension were all high and had the expected sign, corroborating the high correlation among all dimensions.

Correlations between dimension scores and convergent VAS measures were as expected, although the Health Monitoring dimension correlated more highly with the Convenience and Transmission VAS measures. These results may reflect the fact that some sites perform active patient monitoring, personally calling them when any health problem needs to be addressed after automatic transmissions (e.g. due to fluid retention). This being the case, medical monitoring may affect the patient perception of the adequateness of the transmission process and the convenience of the monitoring system. Furthermore, VAS measures were found to be less sensitive to patient differences than dimension scores, behaving as overly holistic measures.

Patient satisfaction, as measured by the Valiosa questionnaire, was shown to have little relation to HRQoL, supporting proper construct validity.

As for known group validity, the different dimensions were shown to be able to discriminate between types of patients. As expected, PM patients were less satisfied with the monitoring system, and perhaps setting up the monitor and making the transmission may even be a burden for them, as we learned from the focus groups. This could be explained because PM patients do not transmit regularly as ICD and CRT patients do because of their medical condition, and hence they might find transmission problems more often that decreases their level of satisfaction with the follow up. The questionnaire dimensions were shown to be sensitive to differences in patient management at different sites, with lower satisfaction for those patients treated more impersonally.

### Benefits and applications of the VALIOSA questionnaire

The present patient satisfaction questionnaire could become a useful instrument in quality-of-care assessment, either being used by clinicians or hospital administrations. In this way, this tool can be used to evaluate patient’s impressions of RM at different times at hospitals and therefore benchmark between different healthcare institutions. The success of telemedicine programs must include patient’s perspectives to understand how well they serve the needs and address the concerns of the patients. Therefore, investigating patients’ impressions with accuracy and precision could help in the design and implementation of telemedicine systems that can improve the quality of healthcare delivery.

## Limitations

Present results may not represent patients using other RM systems without further study. The brand name of the RM system was included in the questionnaire since elder patients showed problems to differentiate between the RM system and the CIED, but replacing the name of the system used in the study with other names should not be a problem. The questionnaire may be adapted to other e-health monitoring systems but important particularities, such as web usability or time to response could be missing. Adaptation to other RM systems may need the inclusion of additional items when special prophylactic measures are customary, such as continuous glucose monitoring systems in insulin pumps for diabetics. Items should be adapted for use of the questionnaire in a telephone interview, since most of them are written in first person.

## Conclusions

In the last years, remote monitoring of patients implanted with CIED has grown as an alternative to in-office follow-up. Rigorous studies have demonstrated its clinical and economic benefits to patients and healthcare institutions. The VALIOSA questionnaire is the first validated questionnaire developed to assess patient satisfaction with RM which has shown good reliability and validity. The questionnaire may be adapted to other cardiac RM systems by changing the name of the system, although maintenance of psychometric properties should be assessed.

## Supplementary information

**Additional file 1.** VALIOSA questionnaire, Spanish version.

**Additional file 2.** VALIOSA Questionnaire, English version.

**Additional file 3.** Additional Materials: Further Psychometric Evidences.

## Data Availability

The datasets generated and analyzed during the current study are not publicly available due to the fact that Ethics Committee approvals were not obtained for sharing of datasets outside of the research team, but are available from the corresponding author on reasonable request.

## Abbreviations

CFA: Confirmatory Factor Analysis
CFI: Comparative fit index
d: Difference
df: Degrees of freedom
F: F statistic
ICC: Intra-class correlation coefficient
RMSEA: Root mean square error of approximation
SD: Standard Deviation
TLI: Tucker-Lewis fit index
