# Supplementary material for: Patient satisfaction with remote monitoring of cardiac implantable electronic devices: the Valiosa questionnaire
Source: BMC Health Serv Res. 2020 Apr 25;20:354. doi: 10.1186/s12913-020-05216-3 (PMC7183665; doi:10.1186/s12913-020-05216-3)
Supplement: Supplementary file 1 — Additional file 1. VALIOSA questionnaire, Spanish version. [file 12913_2020_5216_MOESM1_ESM.docx]

**APPENDIX 1**

CUESTIONARIO DE SATISFACCIÓN CON EL SEGUIMIENTO REMOTO CON EL SISTEMA CARELINK® DE MEDTRONIC (Cuestionario VALIOSA)

En este cuestionario encontrará preguntas sobre su satisfacción con el sistema CareLink® durante el último mes. **Marque una X** en la casilla que mejor describa su opinión sobre el seguimiento remoto de su enfermedad en el último mes. No hay respuestas correctas ni incorrectas. Asegúrese de responder a todas las preguntas.

- Esta sección se refiere a la **Información sobre su enfermedad del corazón.**

|  | No, Nada | Algo | Ni mucho ni poco | Bastante | Sí, Mucho |
| --- | --- | --- | --- | --- | --- |
| 1. Pienso que, actualmente, mi enfermedad del corazón está bien controlada. | 🄋 | ➀ | ➁ | ➂ | ➃ |
| 2. Cuando tengo dudas sobre mi implante sé con quién debo hablar. | 🄋 | ➀ | ➁ | ➂ | ➃ |
| 3. El personal sanitario me ha explicado con detalle cómo ayuda el dispositivo a controlar mi corazón. | 🄋 | ➀ | ➁ | ➂ | ➃ |

- Esta sección hace referencia a la **comodidad del dispositivo.**

|  | No, Nada | Algo | Ni mucho ni poco | Bastante | Sí, Mucho |
| --- | --- | --- | --- | --- | --- |
| 4. Me resulta cómodo usar CareLink®. | 🄋 | ➀ | ➁ | ➂ | ➃ |
| 5. El tiempo entre transmisiones es el adecuado. | 🄋 | ➀ | ➁ | ➂ | ➃ |
| 6. El tiempo que dedico a hacer las trasmisiones es aceptable. | 🄋 | ➀ | ➁ | ➂ | ➃ |

- Esta sección hace referencia al **manejo y el proceso de descarga.**

|  | No, Nada | Algo | Ni mucho ni poco | Bastante | Sí, Mucho |
| --- | --- | --- | --- | --- | --- |
| 7. Me resulta fácil utilizar el sistema CareLink®. | 🄋 | ➀ | ➁ | ➂ | ➃ |
| 8. El entrenamiento que he recibido sobre cómo usar del sistema CareLink® ha sido detallado. | 🄋 | ➀ | ➁ | ➂ | ➃ |
| 9. En ocasiones tengo dudas de si la transmisión se ha completado correctamente. | 🄋 | ➀ | ➁ | ➂ | ➃ |
| 10. El dispositivo CareLink® es fiable y da pocos problemas. | 🄋 | ➀ | ➁ | ➂ | ➃ |
| 11. Cuando hay problemas técnicos el personal responde de manera rápida y me solucionan el problema. | 🄋 | ➀ | ➁ | ➂ | ➃ |
| 12. Tengo problemas para usar CareLink® cuando me desplazo en vacaciones. | 🄋 | ➀ | ➁ | ➂ | ➃ |

- Esta sección se refiere al **seguimiento médico** de su enfermedad.

|  | No, Nada | Algo | Ni mucho ni poco | Bastante | Sí, Mucho |
| --- | --- | --- | --- | --- | --- |
| 13. Confío en el personal sanitario que me trata. | 🄋 | ➀ | ➁ | ➂ | ➃ |
| 14. Usar el sistema CareLink® me hace sentir mejor cuidado por mi médico. | 🄋 | ➀ | ➁ | ➂ | ➃ |
| 15. Estoy satisfecho con la comunicación que mantengo con el personal que hace mi seguimiento en casa. | 🄋 | ➀ | ➁ | ➂ | ➃ |
| 16. Estoy satisfecho con la calidad de las interacciones que realizo con el personal que me sigue en casa. | 🄋 | ➀ | ➁ | ➂ | ➃ |
| 17. El personal que me hace el seguimiento en casa es atento y me responde rápidamente. | 🄋 | ➀ | ➁ | ➂ | ➃ |
| 18. El personal sanitario que me trata revisa con interés los volcados de CareLink®. | 🄋 | ➀ | ➁ | ➂ | ➃ |
| 19. Mi médico utiliza la información de CareLink® en las consultas del hospital. | 🄋 | ➀ | ➁ | ➂ | ➃ |
| 20. La falta de contacto físico usando CareLink® no es un problema para mí. | 🄋 | ➀ | ➁ | ➂ | ➃ |

- Para finalizar, se incluyen unas preguntas acerca de su **opinión general.**

|  | No, Nada | Algo | Ni mucho ni poco | Bastante | Sí, Mucho |
| --- | --- | --- | --- | --- | --- |
| 21. Me siento a gusto usando CareLink®. | 🄋 | ➀ | ➁ | ➂ | ➃ |
| 22. En general, me siento satisfecho con el seguimiento mediante CareLink®. | 🄋 | ➀ | ➁ | ➂ | ➃ |
| 23. Estoy convencido de que el seguimiento con CareLink® es mejor que las visitas presenciales. | 🄋 | ➀ | ➁ | ➂ | ➃ |
| 24. Estoy satisfecho con el programa de seguimiento con CareLink®. | 🄋 | ➀ | ➁ | ➂ | ➃ |
| 25. Hacer el seguimiento con CareLink® me hace sentir más seguro para detectar problemas con mi corazón. | 🄋 | ➀ | ➁ | ➂ | ➃ |
| 26. Utilizar CareLink® me permite estar más en contacto con mis médicos. | 🄋 | ➀ | ➁ | ➂ | ➃ |
| 27. Le recomendaría usar CareLink® a otros pacientes en mi misma situación. | 🄋 | ➀ | ➁ | ➂ | ➃ |
| 28. Utilizar CareLink® me ahorra tiempo en la consulta. | 🄋 | ➀ | ➁ | ➂ | ➃ |
| 29. Utilizando CareLink® tengo que ir menos al hospital. | 🄋 | ➀ | ➁ | ➂ | ➃ |
| 30. El sistema CareLink® me ayuda a manejar mejor mi enfermedad. | 🄋 | ➀ | ➁ | ➂ | ➃ |
