# Supplementary material for: Patient satisfaction with remote monitoring of cardiac implantable electronic devices: the Valiosa questionnaire
Source: BMC Health Serv Res. 2020 Apr 25;20:354. doi: 10.1186/s12913-020-05216-3 (PMC7183665; doi:10.1186/s12913-020-05216-3)
Supplement: Supplementary file 2 — Additional file 2. VALIOSA Questionnaire, English version. [file 12913_2020_5216_MOESM2_ESM.docx]

**APPENDIX 2**

QUESTIONNAIRE ON SATISFACTION WITH REMOTE FOLLOW-UP THROUGH MEDTRONIC CARELINK® NETWORK (VALIOSA Questionnaire)

Within this questionnaire, you will find questions about your satisfaction with CareLink® Network during the past month.

**Mark with an X** the box which better describes your opinion about the remote follow-up of your condition during the past month. There are no correct or incorrect answers. Please make sure that you have answered all questions.

*Note: This is a working translation and psychometric properties may not be ensured.*

- This section is about **Information on your heart condition.**

|  | No, not at all | Slightly | Neutral | Somewhat | Yes, extremely |
| --- | --- | --- | --- | --- | --- |
| 1. I think that my heart condition is currently well controlled. | 🄋 | ➀ | ➁ | ➂ | ➃ |
| 2. When I have doubts about my implant, I know who to speak with. | 🄋 | ➀ | ➁ | ➂ | ➃ |
| 3. The healthcare staff has explained to me in detail how the device helps to control my heart. | 🄋 | ➀ | ➁ | ➂ | ➃ |

- This section is about the **convenience of the device.**

|  | No, not at all | Slightly | Neutral | Somewhat | Yes, extremely |
| --- | --- | --- | --- | --- | --- |
| 4. I find it convenient to use CareLink®. | 🄋 | ➀ | ➁ | ➂ | ➃ |
| 5. The time between transmissions is adequate. | 🄋 | ➀ | ➁ | ➂ | ➃ |
| 6. The time I spend sending transmissions is acceptable. | 🄋 | ➀ | ➁ | ➂ | ➃ |

- This section is about the **management and download process.**

|  | No, not at all | Slightly | Neutral | Somewhat | Yes, extremely |
| --- | --- | --- | --- | --- | --- |
| 7. I find it easy to use the CareLink® system. | 🄋 | ➀ | ➁ | ➂ | ➃ |
| 8. The training I have received on how to use the CareLink® system has been detailed. | 🄋 | ➀ | ➁ | ➂ | ➃ |
| 9. Sometimes I doubt whether the transmission has been completed adequately. | 🄋 | ➀ | ➁ | ➂ | ➃ |
| 10. The CareLink® device is reliable and causes few problems. | 🄋 | ➀ | ➁ | ➂ | ➃ |
| 11. When there are technical problems, the staff will answer immediately and solve the problem. | 🄋 | ➀ | ➁ | ➂ | ➃ |
| 12. I have problems using CareLink® when I go on holidays and need to travel. | 🄋 | ➀ | ➁ | ➂ | ➃ |

- This section is about the **medical follow-up** of your condition.

|  | No, not at all | Slightly | Neutral | Somewhat | Yes, extremely |
| --- | --- | --- | --- | --- | --- |
| 13. I trust the healthcare staff that is treating me. | 🄋 | ➀ | ➁ | ➂ | ➃ |
| 14. Using the CareLink® system makes me feel better cared for by my doctor. | 🄋 | ➀ | ➁ | ➂ | ➃ |
| 15. I am satisfied with the communication I have with the staff conducting my follow-up at home. | 🄋 | ➀ | ➁ | ➂ | ➃ |
| 16. I am satisfied with the quality of the interactions I have with the staff conducting my follow-up at home. | 🄋 | ➀ | ➁ | ➂ | ➃ |
| 17. The staff conducting my follow-up at home is polite and answers immediately. | 🄋 | ➀ | ➁ | ➂ | ➃ |
| 18. The healthcare staff that treats me will review carefully my CareLink® uploads. | 🄋 | ➀ | ➁ | ➂ | ➃ |
| 19. My doctor uses CareLink® information for my hospital visits. | 🄋 | ➀ | ➁ | ➂ | ➃ |
| 20. The lack of physical contact by using CareLink® does not mean a problem for me. | 🄋 | ➀ | ➁ | ➂ | ➃ |

- Finally, some questions are included about your **overall opinion.**

|  | No, not at all | Slightly | Neutral | Somewhat | Yes, extremely |
| --- | --- | --- | --- | --- | --- |
| 21. I feel comfortable using CareLink®. | 🄋 | ➀ | ➁ | ➂ | ➃ |
| 22. Overall, I feel satisfied with the follow-up through CareLink®. | 🄋 | ➀ | ➁ | ➂ | ➃ |
| 23. I am convinced that follow-up with CareLink® is better than face-to-face visits. | 🄋 | ➀ | ➁ | ➂ | ➃ |
| 24. I am satisfied with the CareLink® follow-up program. | 🄋 | ➀ | ➁ | ➂ | ➃ |
| 25. CareLink® follow-up makes me feel more confident in terms of detecting problems with my heart. | 🄋 | ➀ | ➁ | ➂ | ➃ |
| 26. Using CareLink® allows me to keep in closer contact with my doctors. | 🄋 | ➀ | ➁ | ➂ | ➃ |
| 27. I would recommend using CareLink® to other patients in my same situation. | 🄋 | ➀ | ➁ | ➂ | ➃ |
| 28. Using CareLink® saves me time during consultation. | 🄋 | ➀ | ➁ | ➂ | ➃ |
| 29. By using CareLink®, I have to visit the hospital less frequently. | 🄋 | ➀ | ➁ | ➂ | ➃ |
| 30. The CareLink® system helps me to manage my disease better. | 🄋 | ➀ | ➁ | ➂ | ➃ |
