# Supplementary material for: Patient satisfaction with remote monitoring of cardiac implantable electronic devices: the Valiosa questionnaire
Source: BMC Health Serv Res. 2020 Apr 25;20:354. doi: 10.1186/s12913-020-05216-3 (PMC7183665; doi:10.1186/s12913-020-05216-3)
Supplement: Supplementary file 3 — Additional file 3. Additional Materials: Further Psychometric Evidences. [file 12913_2020_5216_MOESM3_ESM.docx]

**APPENDIX 3**

ADDITIONAL MATERIALS: FURTHER PSYCHOMETRIC EVIDENCES.

# Content Validity: Item-Domain congruence

## Unidimensional index

The following table shows the value of the item-domain congruence index proposed by Hambleton & Rovinelli (1977) computed from the content assessment carried out by 9 experts. Values closer to 1 reflect a closer relation of the item with the domain. Values close to -1 indicate that the items is seen as not measuring the domain.

Table 1. Unidimensional item-domain congruence index.

|  | F1 | F2 | F3 | F4 | F5 |
| --- | --- | --- | --- | --- | --- |
| i1 | 0.5 | -0.5 | -0.5 | 0.3 | 0.2 |
| i2 | 0.5 | -0.3 | -0.4 | 0.5 | -0.1 |
| i3 | 0.8 | -0.4 | -0.4 | 0.2 | 0.0 |
| i4 | -0.3 | 0.7 | 0.1 | -0.2 | -0.2 |
| i5 | -0.4 | 0.3 | 0.4 | 0.0 | -0.2 |
| i6 | -0.4 | 0.6 | 0.5 | -0.4 | -0.2 |
| i7 | -0.4 | 0.7 | 0.4 | -0.4 | -0.1 |
| i8 | -0.3 | 0.2 | 0.5 | -0.3 | -0.1 |
| i9 | -0.3 | 0.0 | 0.8 | -0.2 | -0.2 |
| i10 | -0.5 | 0.3 | 0.5 | -0.1 | -0.2 |
| i11 | -0.5 | 0.3 | 0.5 | -0.1 | -0.1 |
| i12 | -0.5 | 0.3 | 0.5 | -0.2 | -0.1 |
| i13 | 0.1 | -0.4 | -0.2 | 0.8 | -0.1 |
| i14 | -0.3 | -0.1 | -0.4 | 0.6 | 0.3 |
| i15 | 0.0 | -0.3 | -0.2 | 0.7 | -0.1 |
| i16 | 0.0 | -0.4 | -0.3 | 0.7 | 0.1 |
| i17 | -0.1 | -0.3 | -0.3 | 0.7 | 0.2 |
| i18 | -0.2 | -0.3 | -0.3 | 0.8 | 0.2 |
| i19 | 0.1 | -0.4 | -0.4 | 0.7 | 0.2 |
| i20 | -0.1 | -0.3 | -0.4 | 0.5 | 0.4 |
| i21 | -0.4 | 0.2 | -0.1 | -0.4 | 0.7 |
| i22 | -0.5 | 0.0 | -0.1 | 0.2 | 0.5 |
| i23 | -0.3 | -0.1 | -0.4 | 0.4 | 0.6 |
| i24 | -0.3 | -0.2 | -0.4 | 0.3 | 0.6 |
| i25 | 0.0 | -0.4 | -0.5 | 0.4 | 0.5 |
| i26 | -0.2 | -0.3 | -0.5 | 0.6 | 0.4 |
| i27 | -0.2 | 0.1 | -0.4 | -0.1 | 0.6 |
| i28 | -0.4 | 0.0 | -0.3 | 0.2 | 0.6 |
| i29 | -0.2 | 0.0 | -0.4 | 0.1 | 0.6 |
| i30 | 0.1 | -0.1 | -0.4 | -0.1 | 0.6 |

## Multidimensional index

The following table shows the value of the item-domain congruence index proposed by Turner & Carlson (2003) computed from the content assessment carried out by 9 experts. Values closer to 1 reflect a closer relation of the item with the multidimensional domain structure. Values close to -1 indicate that the items is seen as not measuring the proposed domains. Columns under the label “theoretical dimensions” show the number of the domain measured by the item. The last column in the table includes the corrected item-total correlation as an additional estimate of item reliability.

Table 2. Multidimensional item-domain congruence index.

|  | **Congruence Index** | **Theoretical**  **Dimensions** | | **Corrected  Item-Total Correlation** |
| --- | --- | --- | --- | --- |
|  |  | **I** | **II** |  |
| i1 | 0.81 | 1 | 4 | .278 |
| i2 | 0.95 | 1 | 4 | .461 |
| i3 | 0.76 | 1 | 4 | .519 |
| i4 | 0.68 | 2 | 3 | .519 |
| i5 | 0.66 | 2 | 3 | .665 |
| i6 | 1.03 | 2 | 3 | .643 |
| i7 | 0.87 | 3 | 2 | .411 |
| i8 | 0.61 | 3 | 2 | .314 |
| i9 | 0.61 | 3 | 2 | .213 |
| i10 | 0.79 | 3 | 2 | .419 |
| i11 | 0.72 | 3 | 2 | .303 |
| i12 | 0.80 | 3 | 2 | .332 |
| i13 | 0.88 | 4 | * | .379 |
| i14 | 0.74 | 4 | * | .422 |
| i15 | 0.84 | 4 | * | .571 |
| i16 | 0.80 | 4 | * | .565 |
| i17 | 0.84 | 4 | * | .495 |
| i18 | 0.83 | 4 | * | .415 |
| i19 | 0.82 | 4 | * | -.071 |
| i20 | 0.63 | 4 | * | .551 |
| i21 | 0.78 | 5 | * | .525 |
| i22 | 0.55 | 5 | 4 | .664 |
| i23 | 0.87 | 5 | 4 | .632 |
| i24 | 0.79 | 5 | 4 | .487 |
| i25 | 0.98 | 5 | 4 | .546 |
| i26 | 0.99 | 5 | 4 | .470 |
| i27 | 0.77 | 5 | * | .368 |
| i28 | 0.72 | 5 | 4 | .539 |
| i29 | 0.63 | 5 | 4 | .689 |
| i30 | 0.57 | 5 | 4 | .704 |

* Not needed.

# Exploratory and Confirmatory Factor Analysis by Dimension

The following tables show the results on the Exploratory Factor Analysis of each dimension items and the standardized estimates of the Confirmatory Factor Analysis.

## Information about the cardiac disease

Table 3. Eigenvalues and Accounted Variance for Information dimension (1 factor solution)

| **Total Variance Explained** | | | | | | |
| --- | --- | --- | --- | --- | --- | --- |
| Component | Initial Eigenvalues | | | Extraction Sums of Squared Loadings | | |
|  | Total | % of Variance | Cumulative % | Total | % of Variance | Cumulative % |
| 1 | 1.685 | 56.173 | 56.173 | 1.685 | 56.173 | 56.173 |
| 2 | .825 | 27.484 | 83.657 |  |  |  |
| 3 | .490 | 16.343 | 100.000 |  |  |  |
| Extraction Method: Principal Component Analysis. | | | | | | |

Table 4. Exploratory Factor loadings on the Information dimension

| **Component Matrix^a^** | |
| --- | --- |
|  | Component |
|  | 1 |
| 1. I think that my heart condition is currently well controlled. | .589 |
| 2. When I have doubts about my implant, I know who to speak with. | .807 |
| 3. The healthcare staff has explained to me in detail how the device helps to control my heart. | .828 |
| Extraction Method: Principal Component Analysis. | |
| a. 1 components extracted. | |


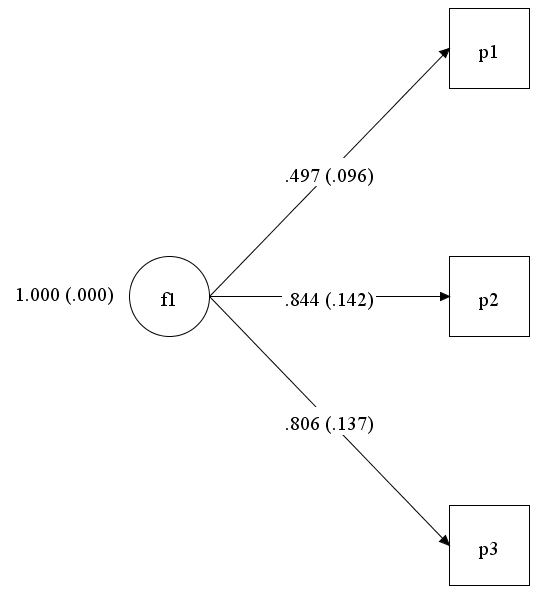


Figure 1. Confirmatory Standardized factor loading estimates (standard errors) for the Infomrmation dimension

## Transmitter convenience

Table 5. Eigenvalues and Accounted Variance for Convenience dimension (1 factor solution)

| **Total Variance Explained** | | | | | | |
| --- | --- | --- | --- | --- | --- | --- |
| Component | Initial Eigenvalues | | | Extraction Sums of Squared Loadings | | |
|  | Total | % of Variance | Cumulative % | Total | % of Variance | Cumulative % |
| 1 | 2.073 | 69.087 | 69.087 | 2.073 | 69.087 | 69.087 |
| 2 | .585 | 19.486 | 88.574 |  |  |  |
| 3 | .343 | 11.426 | 100.000 |  |  |  |
| Extraction Method: Principal Component Analysis. | | | | | | |

Table 6. Exploratory Factor loadings on the Convenience dimension

| **Component Matrix^a^** | |
| --- | --- |
|  | Component |
|  | 1 |
| 4. I find it convenient to use CareLink. | .761 |
| 5. The time between transmissions is adequate. | .869 |
| 6. The time I spend sending transmissions is acceptable. | .859 |
| Extraction Method: Principal Component Analysis. | |
| a. 1 components extracted. | |


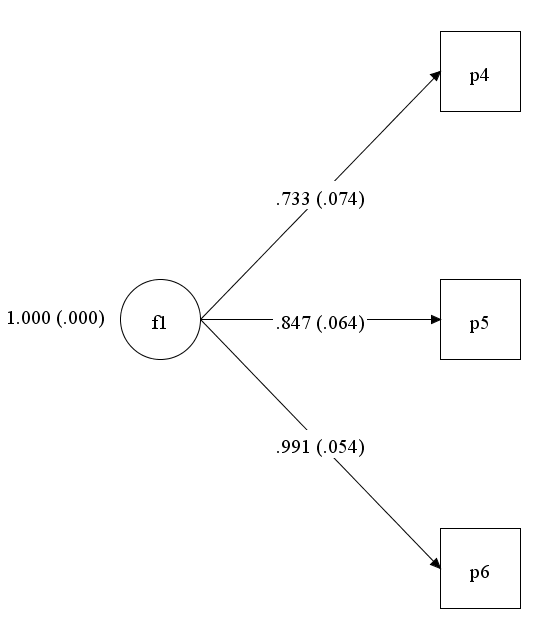


Figure 2. Confirmatory Standardized factor loading estimates (standard errors) for the Convenience dimension

## Transmission process and handling

Table 7. Eigenvalues and Accounted Variance for Transmission Process dimensions (2 factor solution Promax rotation)

| **Total Variance Explained** | | | | | | | |
| --- | --- | --- | --- | --- | --- | --- | --- |
| Component | Initial Eigenvalues | | | Extraction Sums of Squared Loadings | | | Rotation Sums of Squared Loadings^a^ |
|  | Total | % of Variance | Cumulative % | Total | % of Variance | Cumulative % | Total |
| 1 | 2.135 | 35.583 | 35.583 | 2.135 | 35.583 | 35.583 | 1.909 |
| 2 | 1.182 | 19.699 | 55.282 | 1.182 | 19.699 | 55.282 | 1.660 |
| 3 | .870 | 14.502 | 69.784 |  |  |  |  |
| 4 | .806 | 13.429 | 83.214 |  |  |  |  |
| 5 | .644 | 10.736 | 93.950 |  |  |  |  |
| 6 | .363 | 6.050 | 100.000 |  |  |  |  |
| Extraction Method: Principal Component Analysis. | | | | | | | |
| a. When components are correlated. sums of squared loadings cannot be added to obtain a total variance. | | | | | | | |

Table 8. Exploratory Factor loadings on the Transmission Process dimension (1 factor solution)

| **Component Matrix^a^** | |
| --- | --- |
|  | Component |
|  | 1 |
| 7. I find it easy to use the CareLink system. | .742 |
| 8. The training I have received on how to use the CareLink system has been detailed. | .623 |
| 9. Sometimes I doubt whether the transmission has been completed adequately. | -.412 |
| 10. The CareLink device is reliable and causes few problems. | -.595 |
| 11. When there are technical problems, the staff will answer immediately and solve the problem. | .565 |
| 12. I have problems using CareLink when I go on holidays and need to travel. | -.593 |
| Extraction Method: Principal Component Analysis. | |
| a. 1 components extracted. | |

Table 9. Exploratory Factor loadings on the Transmission Process dimensions (2 factor solution Promax rotation)

| **Component Matrix^a^** | | |
| --- | --- | --- |
|  | Component | |
|  | 1 | 2 |
| 7. I find it easy to use the CareLink system. | .826 | -.269 |
| 8. The training I have received on how to use the CareLink system has been detailed. | .768 | -.118 |
| 9. Sometimes I doubt whether the transmission has been completed adequately. | -.061 | .724 |
| 10. The CareLink device is reliable and causes few problems. | -.239 | .829 |
| 11. When there are technical problems, the staff will answer immediately and solve the problem. | .636 | -.193 |
| 12. I have problems using CareLink when I go on holidays and need to travel. | -.414 | .571 |
| Extraction Method: Principal Component Analysis. | | |
| a. 2 components extracted. | | |

Table 10. Coorlation between Transmission Process dimensions (2 factor solution Promax rotation)

| **Component Correlation Matrix** | | |
| --- | --- | --- |
| Component | 1 | 2 |
| 1 | 1.000 | -.275 |
| 2 | -.275 | 1.000 |
|  | | |


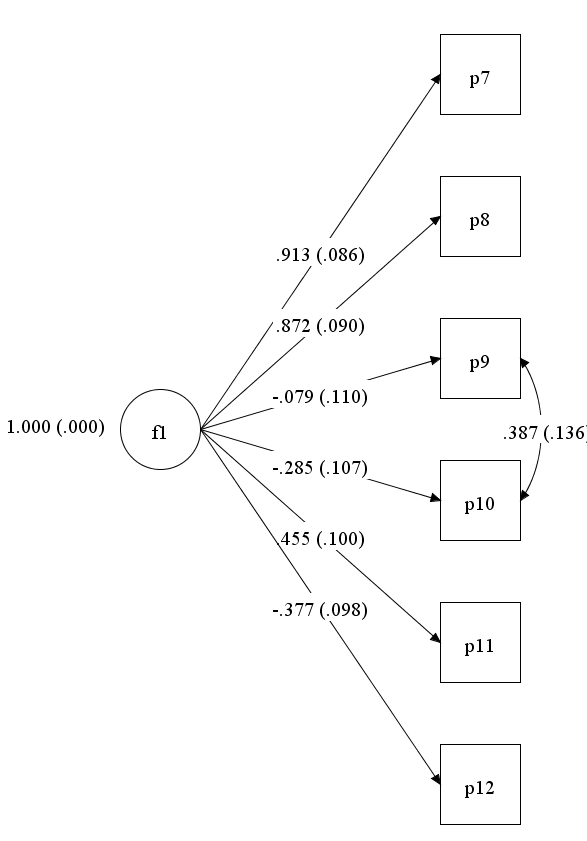


Figure 3. Confirmatory Standardized factor loading estimates (standard errors) for the Transmission dimension

## Medical follow-up

Table 11. Eigenvalues and Accounted Variance for Information dimension (1 factor solution)

| **Total Variance Explained** | | | | | | |
| --- | --- | --- | --- | --- | --- | --- |
| Component | Initial Eigenvalues | | | Extraction Sums of Squared Loadings | | |
|  | Total | % of Variance | Cumulative % | Total | % of Variance | Cumulative % |
| 1 | 2.947 | 42.095 | 42.095 | 2.947 | 42.095 | 42.095 |
| 2 | 1.037 | 14.816 | 56.911 |  |  |  |
| 3 | .851 | 12.153 | 69.064 |  |  |  |
| 4 | .674 | 9.624 | 78.688 |  |  |  |
| 5 | .595 | 8.506 | 87.194 |  |  |  |
| 6 | .566 | 8.091 | 95.285 |  |  |  |
| 7 | .330 | 4.715 | 100.000 |  |  |  |
| Extraction Method: Principal Component Analysis. | | | | | | |

Table 12. Exploratory Factor loadings on the Follow-up dimension

| **Component Matrix^a^** | |
| --- | --- |
|  | Component |
|  | 1 |
| 13. I trust the healthcare staff that is treating me. | .571 |
| 14. Using the CareLink system makes me feel better cared for by my doctor. | .663 |
| 15. I am satisfied with the communication I have with the staff conducting my follow-up at home. | .821 |
| 16. I am satisfied with the quality of the interactions I have with the staff conducting my follow-up at home. | .795 |
| 17. The staff conducting my follow-up at home is polite and answers immediately. | .674 |
| 18. The healthcare staff that treats me will review carefully my CareLink uploads. | .640 |
| 19. My doctor uses CareLink information for my hospital visits. | .099 |
| Extraction Method: Principal Component Analysis. | |
| a. 1 components extracted. | |


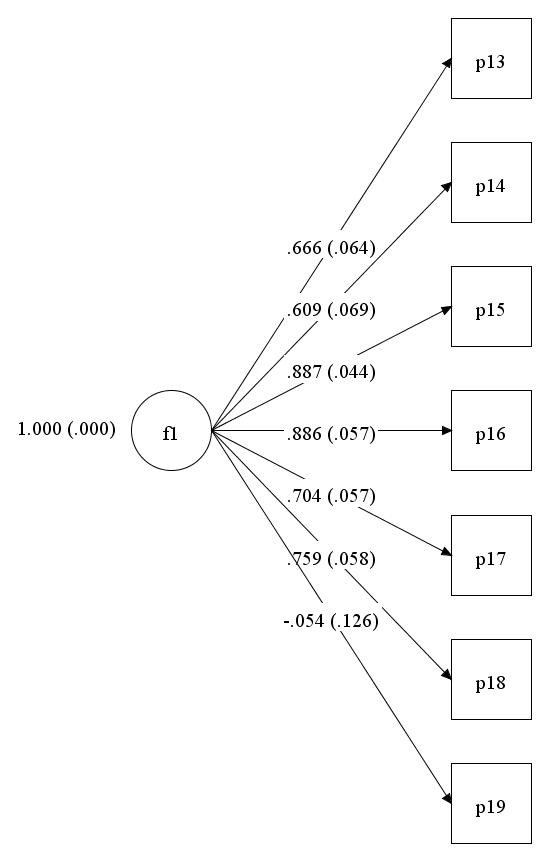


Figure 4. Confirmatory Standardized factor loading estimates (standard errors) for the Follow-up dimension

## General opinions and benefits

Table 13. Eigenvalues and Accounted Variance for Information dimension (1 factor solution)

| **Total Variance Explained** | | | | | | |
| --- | --- | --- | --- | --- | --- | --- |
| Component | Initial Eigenvalues | | | Extraction Sums of Squared Loadings | | |
|  | Total | % of Variance | Cumulative % | Total | % of Variance | Cumulative % |
| 1 | 4.977 | 45.246 | 45.246 | 4.977 | 45.246 | 45.246 |
| 2 | 1.318 | 11.986 | 57.232 |  |  |  |
| 3 | .883 | 8.025 | 65.257 |  |  |  |
| 4 | .741 | 6.738 | 71.996 |  |  |  |
| 5 | .614 | 5.578 | 77.574 |  |  |  |
| 6 | .604 | 5.492 | 83.065 |  |  |  |
| 7 | .466 | 4.236 | 87.301 |  |  |  |
| 8 | .414 | 3.762 | 91.063 |  |  |  |
| 9 | .363 | 3.299 | 94.361 |  |  |  |
| 10 | .343 | 3.116 | 97.477 |  |  |  |
| 11 | .277 | 2.523 | 100.000 |  |  |  |
| Extraction Method: Principal Component Analysis. | | | | | | |

Table14. Exploratory Factor loadings on the General Oppinions dimension (1 factor solution)

| **Component Matrix^a^** | |
| --- | --- |
|  | Component |
|  | 1 |
| 21. I feel comfortable using CareLink. | .696 |
| 22. Overall, I feel satisfied with the follow-up through CareLink. | .649 |
| 23. I am convinced that follow-up with CareLink is better than face-to-face visits. | .799 |
| 24. I am satisfied with the CareLink follow-up program. | .750 |
| 25. CareLink follow-up makes me feel more confident in terms of detecting problems with my heart. | .622 |
| 26. Using CareLink allows me to keep in closer contact with my doctors. | .654 |
| 27. I would recommend using CareLink to other patients in my same situation. | .515 |
| 28. Using CareLink saves me time during consultation. | .396 |
| 29. By using CareLink, I have to visit the hospital less frequently. | .570 |
| 30. The CareLink system helps me to manage my disease better. | .799 |
| 21. I feel comfortable using CareLink. | .819 |
| Extraction Method: Principal Component Analysis. | |
|  | |


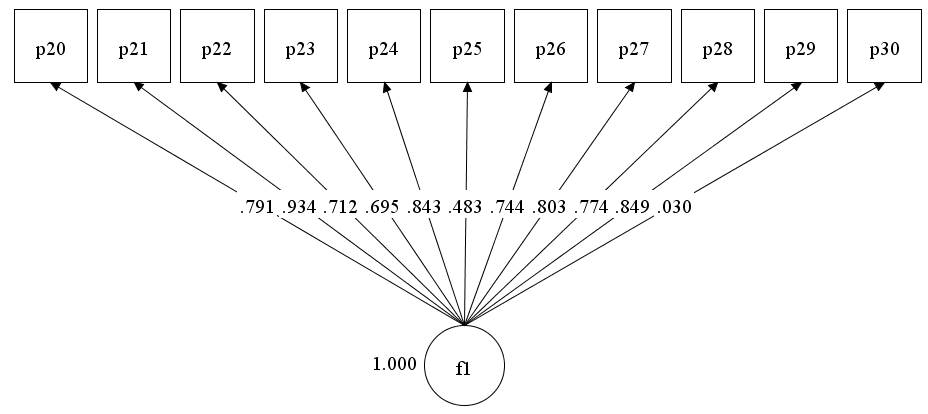


Figure 5. Confirmatory Standardized factor loading estimates (standard errors), Overall Oppinions dimension


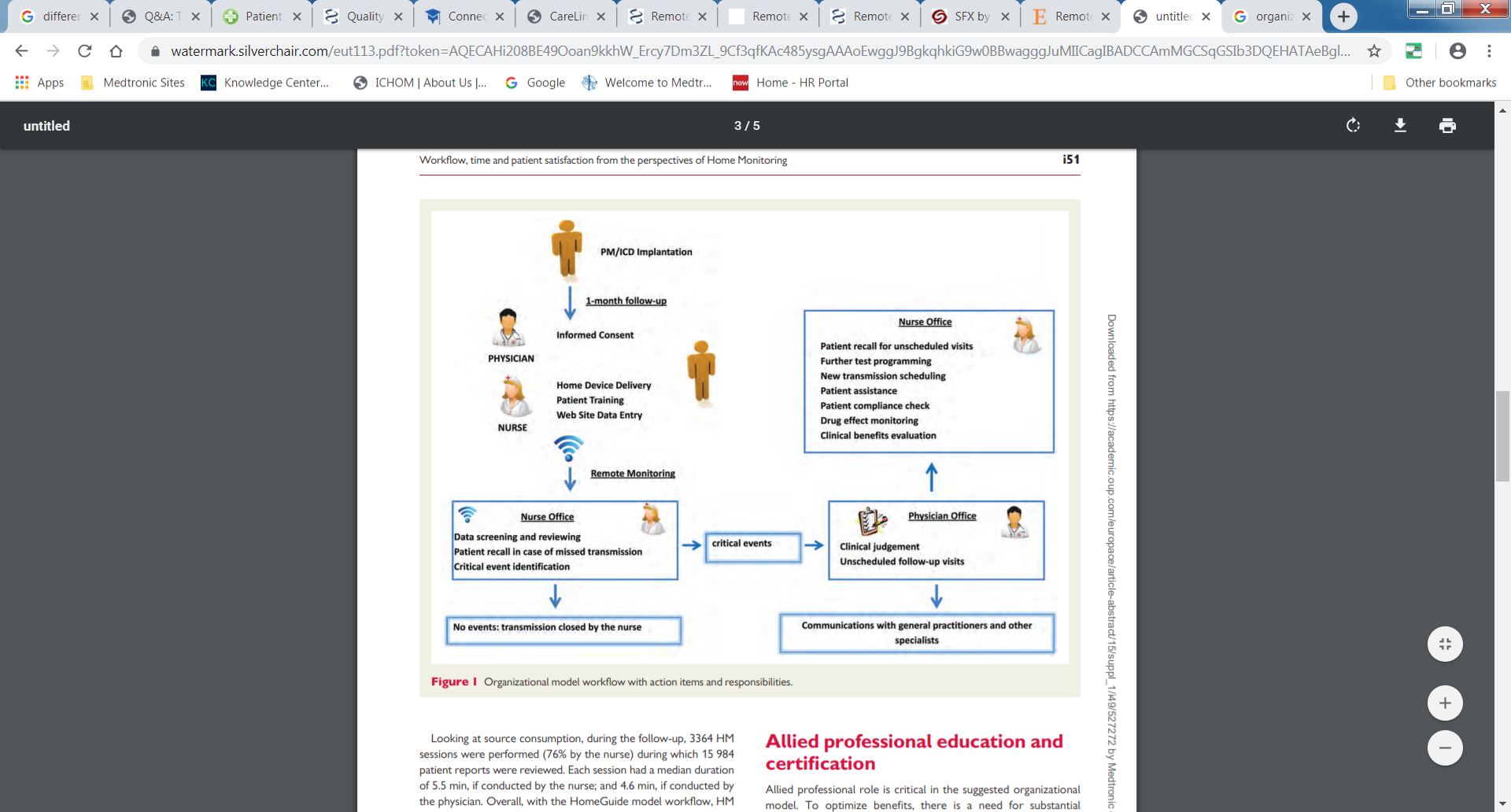


Figure 6. The organizational model workflow called “primary nursing” with action items and responsibilities. Adapted from Ricci, L. Morichelli, Workflow, time and patient satisfaction from the perspectives of home monitoring, Europace 15 (Suppl. 1) (2013) i49–i53.
